# Supplementary material for: The role of Notch signaling in endometrial mesenchymal stromal/stem-like cells maintenance
Source: Commun Biol. 2022 Oct 7;5:1064. doi: 10.1038/s42003-022-04044-x (PMC9547015; doi:10.1038/s42003-022-04044-x)
Supplement: Supplementary file 2 — Supplementary information [file 42003_2022_4044_MOESM2_ESM.pdf]

Title: The Role of Notch Signaling in Endometrial Mesenchymal Stromal/Stem-like Cells Maintenance

Supplementary Figure

Supplementary Figure 1. The percentage of $CD146^{+}CD140b^{+}NICD^{+}$ in endometrial stromal cells after Notch signals activation or inhibition.

Supplementary Figure 2. The effect of Notch signaling on eMSC apoptosis.

Supplementary Figure 3. The percentage of $CD146^{+}CD140b^{+}ABC^{+}$ in endometrial stromal cells after Notch signals activation or inhibition.

Supplementary Figure 4. Establishment and histological analysis of mouse menstrual-like model.

Supplementary Figure 5. Uncropped scans of western blots.

Supplementary Figure 6. Uncropped scans of western blots.

Supplementary Table

Supplemental Table S1. Pathological Characteristic of Full Thickness Endometrial.

Supplementary Table S2. List of primary antibodies used for immunohistochemistry (IHC) and immunofluorescent (IF) staining.

Supplemental Table S3. List of secondary antibodies used for immunohistochemistry (IHC) and immunofluorescent (IF) staining.

Supplemental Table S4. List of primary and secondary antibodies used for western blotting.

Supplemental Table S5. Taqman probes used for qPCR.

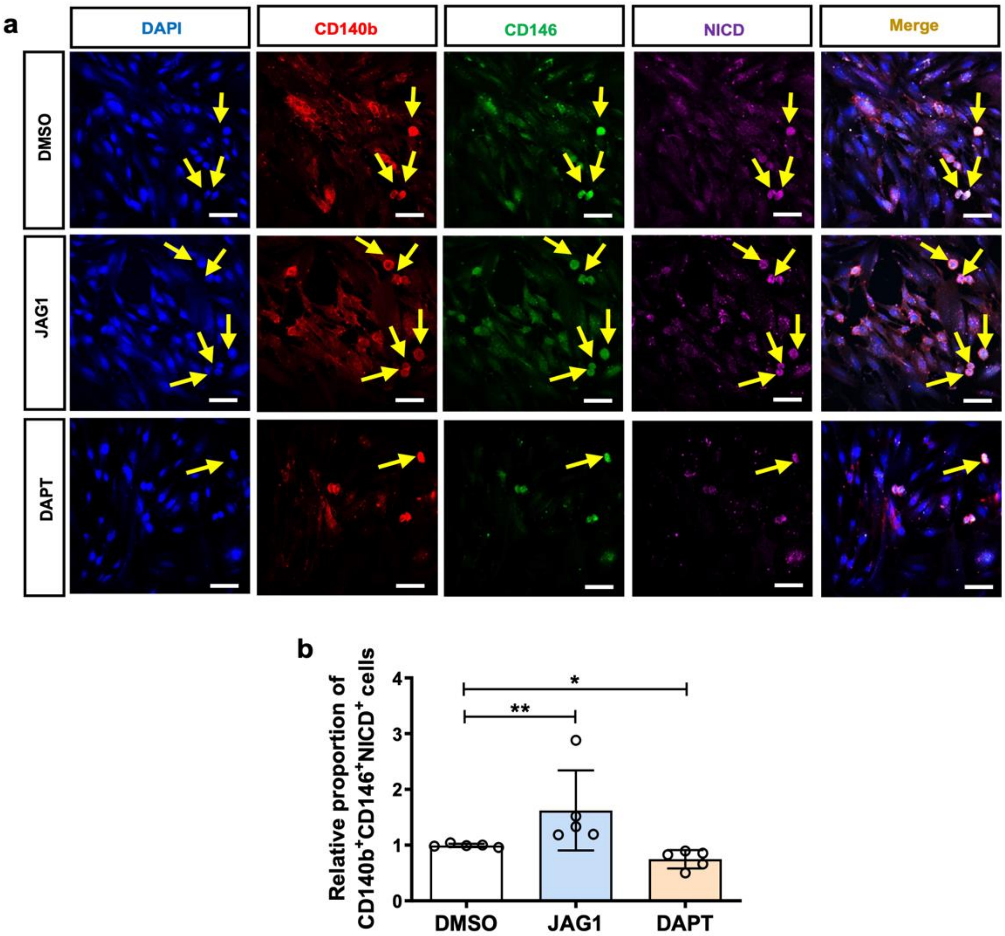

Supplementary Figure 1. **The percentage of CD146⁺CD140b⁺NICD⁺ in endometrial stromal cells after Notch signals activation or inhibition.**

(a) Representative images showing the triple staining of CD146⁺CD140b⁺NICD⁺ (yellow arrows) in endometrial stromal cells after Notch signals activation or inhibition. Scale bar: 50 µm. **(b)** Quantitative analysis of CD140b⁺CD146⁺NICD⁺ cells after Notch signals activation or inhibition. Results are presented as mean ± SD; n=5; *P < .05; **P < .01. Statistical analysis was performed using One-way ANOVA followed by Tukey's test for multiple group comparison.

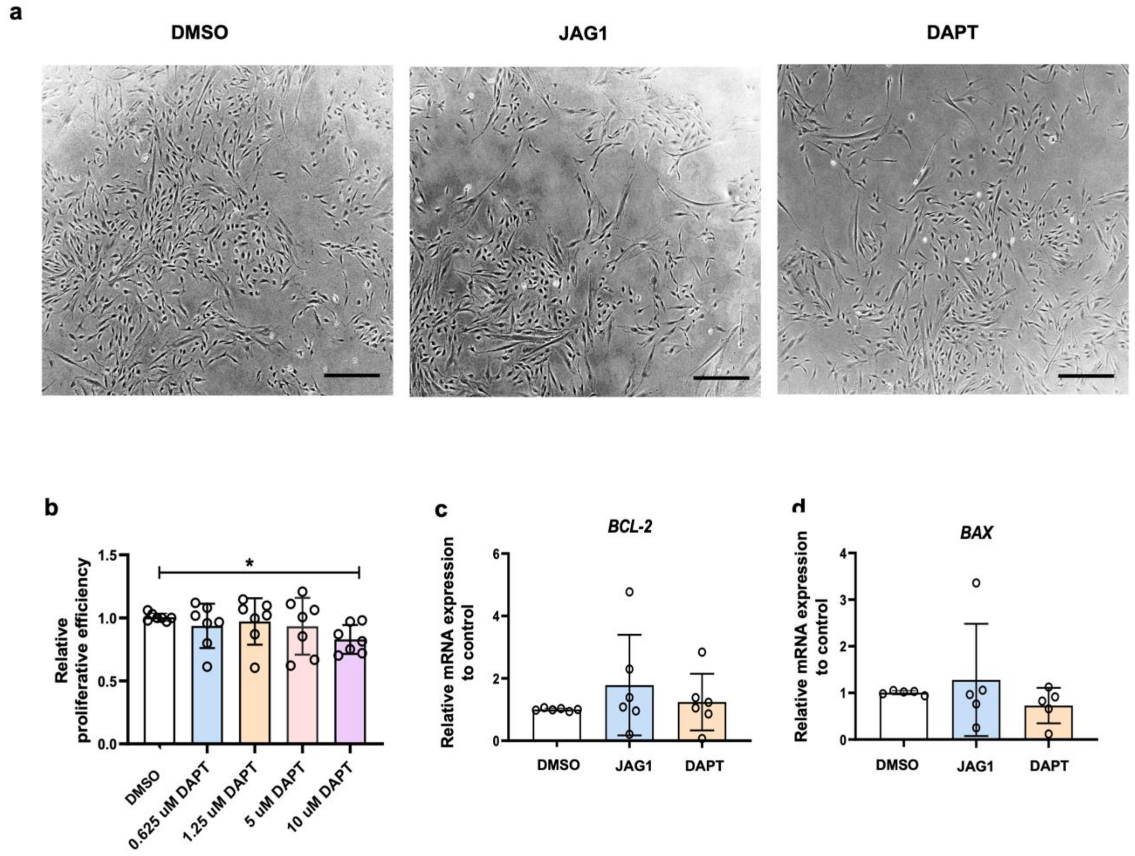

Supplementary Figure 2. The effect of Notch signaling on eMSC apoptosis.
(a) Representative phase-contrast images of eMSC cultured under different condition for 7 days, scale bar: 100 μm. **(b)** The relative proliferative efficiency of eMSC under different DAPT concentration (n=7). **(c)** The relative gene expression of anti-apoptosis marker *BCL-2* in eMSC after Notch signals activation or inhibition (n=6). **(d)** The relative gene expression of apoptosis marker *BAX* in eMSCs after Notch signals activation or inhibition (n=5). Results are presented as mean ± SD. *P < .05. Statistical analysis was performed using One-way ANOVA followed by Tukey's test for multiple group comparison.

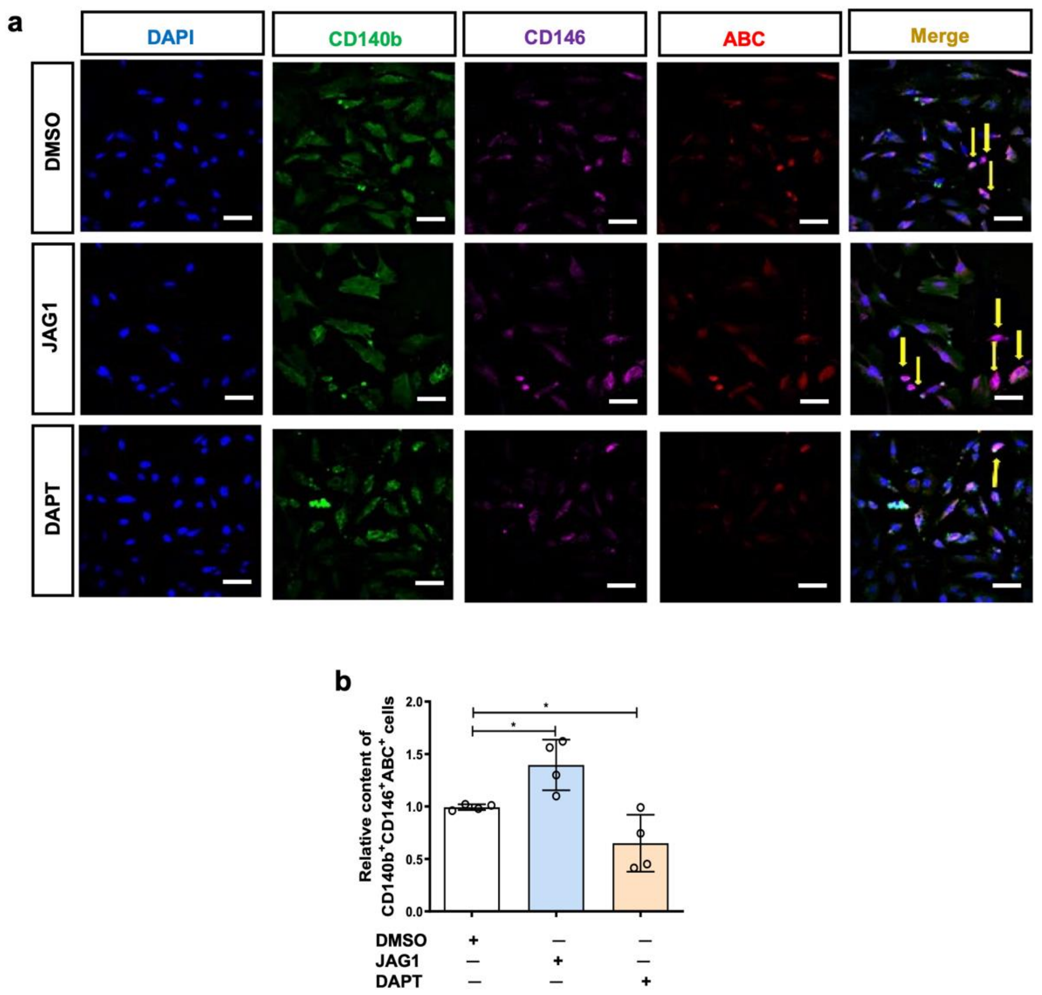

Supplementary Figure 3. The percentage of CD146$^+$CD140b$^+$ABC$^+$ in endometrial stromal cells after Notch signals activation or inhibition.

(a) Representative images showing the triple staining of CD146$^+$CD140b$^+$ABC$^+$ (yellow arrows) in endometrial stromal cells after Notch signals activation or inhibition. scale bar: 50 μm. **(b)** Quantitative analysis of CD146$^+$CD140b$^+$ABC$^+$ endometrial stromal cells after Notch signals activation or inhibition (n=4). Results are presented as mean ± SD. *P < .05. Statistical analysis was performed using One-way ANOVA followed by Tukey's test for multiple group comparison. Abbreviation: ABC: active β-catenin.

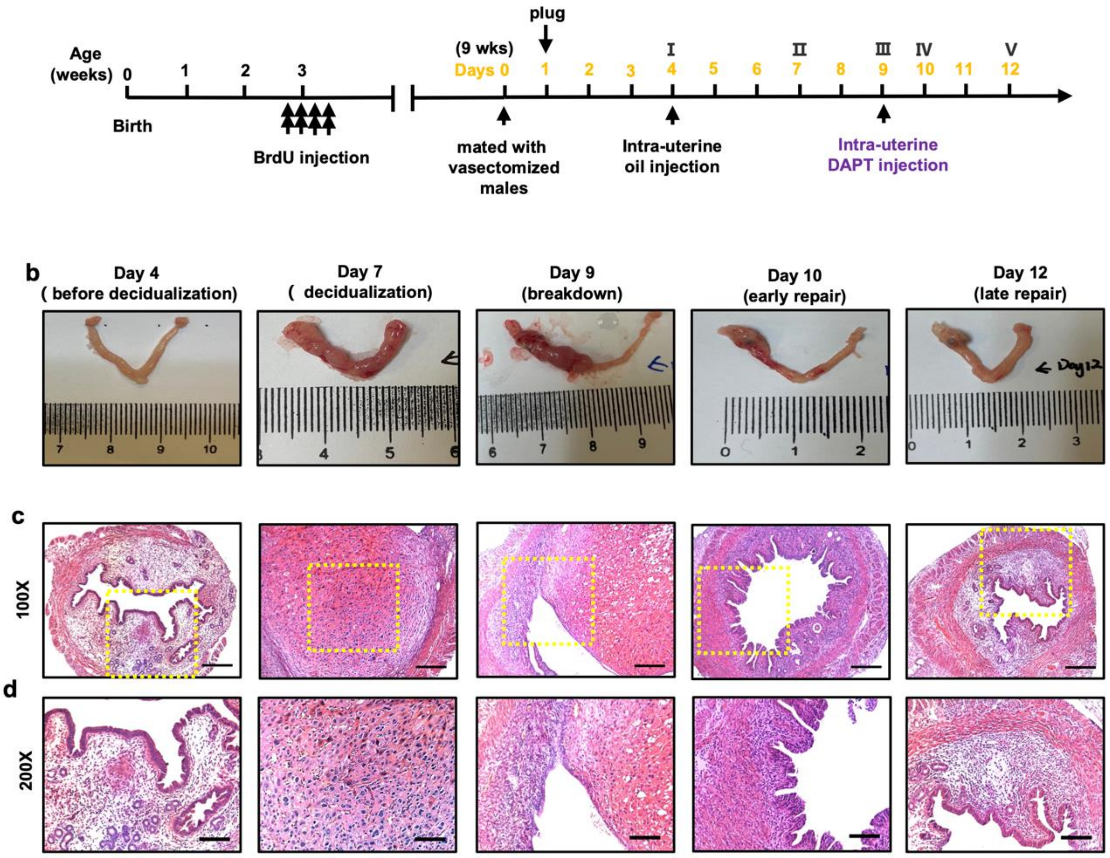

Supplementary Figure 4. Establishment and histological analysis of mouse menstrual-like model.

(a) Timeline of mouse menstrual-like model. Prepubertal (day 19) C57BL/6J female mice were pulse labeled with BrdU (50 mg/g of body weight) twice daily for 4 days. After chasing for 5-6 weeks, female mice were mated with vasectomized male mice (day 0). Pseudopregnant female mice were identified by vaginal plugs on the next day (day 1). On day 4, endometrial decidualization was induced by intrauterine injection of sesame oil. Tissue was collected at day 4 (before decidualization, denoted by I), day 7 (decidualization, denoted by II), day 9 (breakdown, denoted by III), day 10 (early repair, as denoted by IV), and day 12 (late repair, denoted by V). **(b)** Macroscopic analysis of uteri at indicated time points. The right uterine horn severed as control. **(c)** Microscopic analysis of H&E-stained uteri at indicated time points, scale bar: 100 μm. **(d)** High magnification images of the yellow square shown in C, scale bar: 50 μm. n = 3–5 per group.

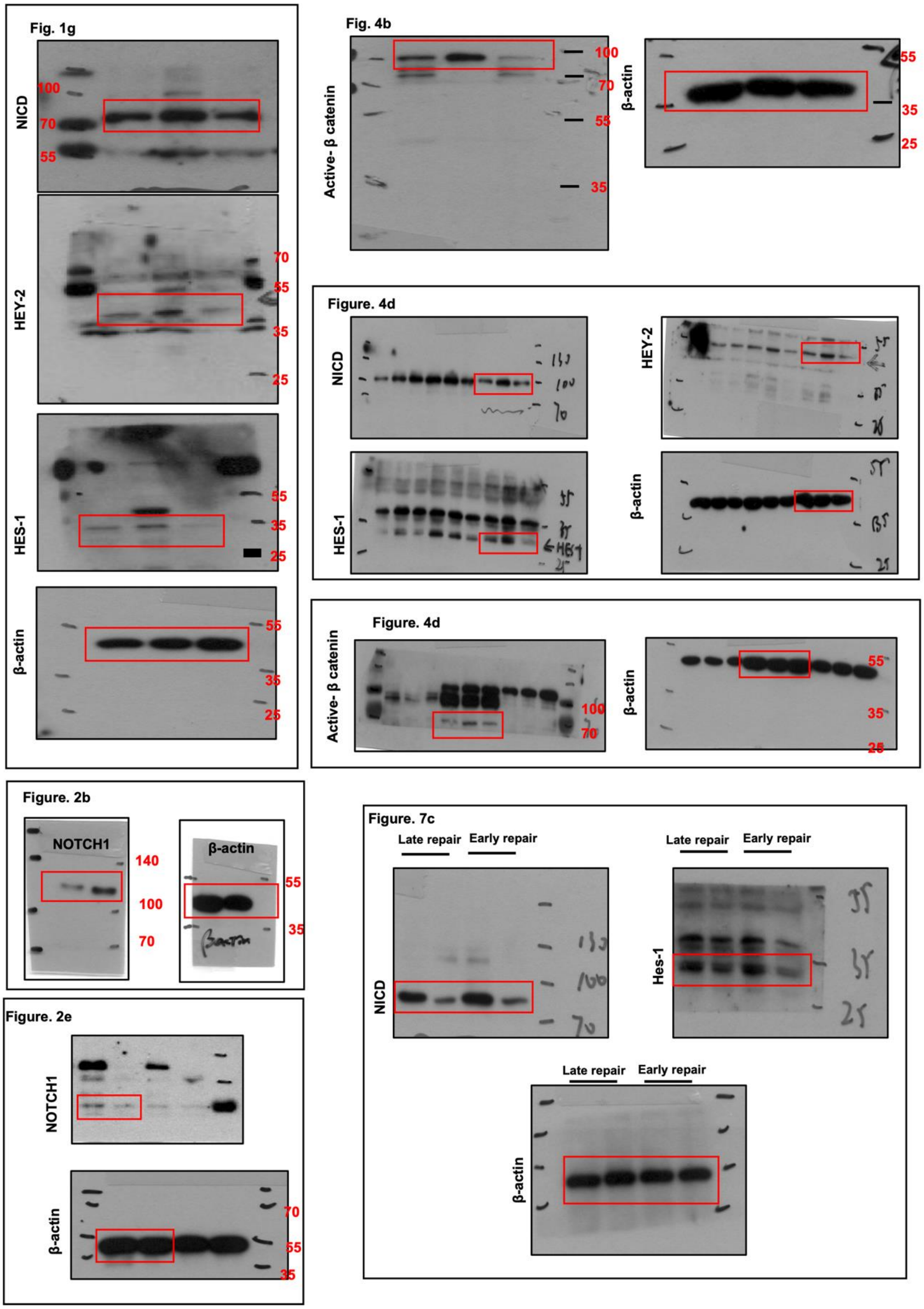

Supplementary Figure 5. Uncropped scans of western blots. The cropped region is highlighted with the red boxes.

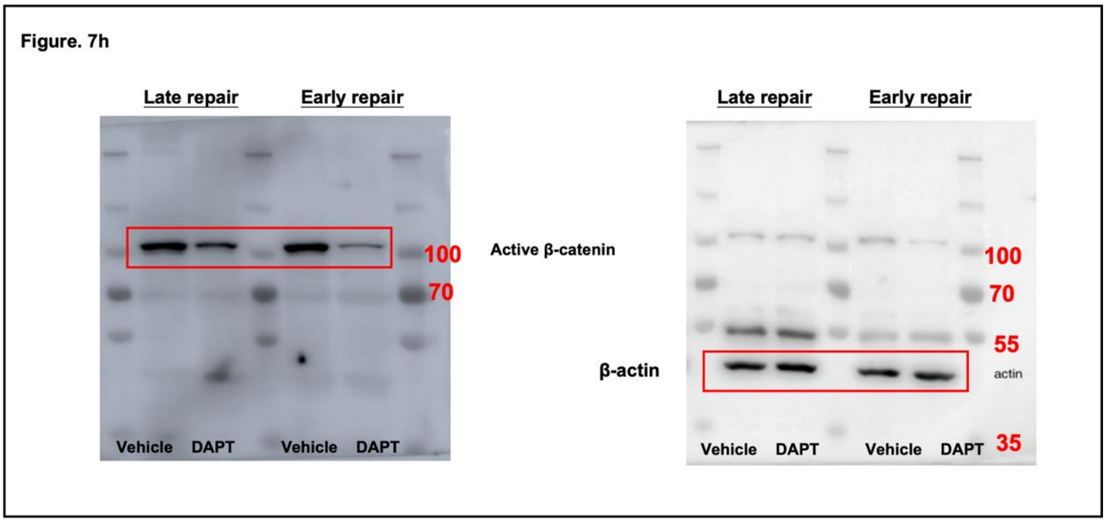

Figure S6. Uncropped scans of western blots. The cropped region is highlighted with the red boxes.

Supplementary Table S1. Pathological Characteristic of Full Thickness Endometrial

	Age	Menstrual Phase	Pathology
1	45	proliferative	leiomyomas
2	47	proliferative	leiomyomas
3	48	proliferative	leiomyomas
4	43	proliferative	leiomyomas
5	47	proliferative	leiomyomas
6	47	proliferative	leiomyomas
7	50	proliferative	leiomyomas
8	41	proliferative	leiomyomas
9	47	proliferative	leiomyomas
10	45	proliferative	leiomyomas
11	46	proliferative	adenomyosis
12	46	proliferative	leiomyomas
13	43	proliferative	adenomyosis
14	46	proliferative	leiomyomas
15	48	proliferative	adenomyosis
16	47	proliferative	leiomyomas
17	49	secretory	adenomyosis
18	52	secretory	leiomyomas
19	45	secretory	leiomyomas
20	44	secretory	leiomyomas
21	48	secretory	leiomyomas
22	44	secretory	leiomyomas
23	49	secretory	leiomyomas
24	44	secretory	leiomyomas
25	47	secretory	leiomyomas
26	46	secretory	adenomyosis
27	48	secretory	leiomyomas

Supplementary Table S2. List of primary antibodies used for immunohistochemistry (IHC) and immunofluorescent (IF) staining. Related to the results in Figure 1f, Figure 2, Figure 4c, Figure 5, Figure 6, Figure 8, Figure 9, Supplementary Figure 1 and Supplementary Figure 3.

Primary Antibody	Source	Dilution
**Active β-catenin:** mouse monoclonal to active β-actin	Millipore	1:100
**BrdU:** sheep polyclonal to BrdU	Abcam	1:500
**CD146:** mouse polyclonal to CD146	Novus	1:100
**CD146:** rabbit polyclonal to CD146	Abcam	1:100
**CD140b:** goat polyclonal to CD140b	Abcam	1:100
**Ki67:** rabbit polyclonal to ki-67	Abcam	1:500
**Notch1:** rabbit polyclonal to Notch1	Biorbyt	1:100
**Notch1 Intracellular domain (NICD):** rabbit polyclonal to NICD	Millipore	1:100
**Jagged1**: rabbit polyclonal to Jagged1	CST	1:200
**DLL4:** rabbit polyclonal anti-DLL4	Novus	1:200

Supplemental Table S3. List of secondary antibodies used for immunohistochemistry (IHC) and immunofluorescent (IF) staining. Related to the results in Figure 1f, Figure 2, Figure 4c, Figure 5, Figure 6, Figure 8, Figure 9, Supplementary Figure 1 and Supplementary Figure 3.

Antibody	Conjugation	Source	Dilution
**Donkey anti-rabbit**	Alexa Fluor 647	Life technologies	1:200
**Rabbit anti-mouse**	Alexa Fluor 488	Life technologies	1:200
**Donkey anti-goat**	Alexa Fluor 555	Life technologies	1:200
**Rabbit anti-goat**	Alexa Fluor 488	Life technologies	1:200
**Donkey anti-mouse**	Alexa Fluor 568	Life technologies	1:200
**Donkey anti-sheep**	Alexa Fluor 555	Life technologies	1:200
**Donkey anti-sheep**	Biotin	Abcam	1:400

Supplemental Table S4. List of primary and secondary antibodies used for western blotting. Related to the results in Figure 1, Figure 2, Figure 4, and Figure 7.

Primary Antibody (Source, Dilution)	Secondary Antibody (Source, Dilution)
**Nocth1**: rabbit polyclonal to Nocth1 (Biorbyt, 1:500)	Rabbit horseradish peroxidase (GE Healthcare, 1:5000)
**Notch1 Intracellular domain (NICD)**: rabbit polyclonal to NICD (Millipore, 1:1000)	Rabbit horseradish peroxidase (GE Healthcare, 1:5000)
**Active β-catenin**: rabbit monoclonal to active β-actin (CST,1:1000)	Rabbit horseradish peroxidase (GE Healthcare, 1:5000)
**HEY-2**: rabbit polyclonal to HEY-2 (Millipore, 1:1000)	Rabbit horseradish peroxidase (GE Healthcare, 1:5000)
**HES-1**: rabbit polyclonal to HES-1 (Millipore, 1:500)	Rabbit horseradish peroxidase (GE Healthcare, 1:5000)
**β-actin:** mouse monoclonal beta actin (Sigma, 1:5000)	Mouse horseradish peroxidase (GE Healthcare, 1:5000)

Supplemental Table S5. Taqman probes used for qPCR. Related to RT- qPCR results in Figure 1, Figure 2, Figure 3, Figure 7 and Supplementary Figure 2.

**Gene Name *(Gene Symbol)***	**Taqman Probes**
Hairy and enhancer of split-1 (*HES1)*	Hs00172878_m1
Hairy/enhancer-of-split related with YRPW motif protein 1 *(HEY1)*	Hs01114113_m1
Hairy/enhancer-of-split related with YRPW motif protein 2 *(HEY2)*	Hs01012057_m1
Hairy/enhancer-of-split related with YRPW motif protein L *(HEYL)*	Hs01113778_m1
Notch receptor 1 *(Notch1)*	Hs01062014_m1
Notch receptor 2 *(Notch2)*	Hs01050702_m1
Notch receptor 3 *(Notch3)*	Hs00166432_m1
CDKN1A	Hs00355782_m1
CDKN1B	Hs00153277_m1
CCNA2	Hs00996788_m1
CCNE2	Hs00180319_m1
CCND1	Hs00765553_m1
GOS2	Hs00377852_m1
MKI67	Hs00757500_m1
BIRC5	Hs04194392_m1
BAX	Hs04986394_s1
BCL-2	Hs04986394_s1
