# Supplementary material for: The role of Notch signaling in endometrial mesenchymal stromal/stem-like cells maintenance
Source: Commun Biol. 2022 Oct 7;5:1064. doi: 10.1038/s42003-022-04044-x (PMC9547015; doi:10.1038/s42003-022-04044-x)
Supplement: Supplementary file 3 — Description of Additional Supplementary Files [file 42003_2022_4044_MOESM3_ESM.pdf]

Description of Additional Supplementary Files

File name: Supplementary Data
Description: The data that support the findings of this study.
