# Supplementary material for: The role of Notch signaling in endometrial mesenchymal stromal/stem-like cells maintenance
Source: Commun Biol. 2022 Oct 7;5:1064. doi: 10.1038/s42003-022-04044-x (PMC9547015; doi:10.1038/s42003-022-04044-x)
Supplement: Supplementary file 5 — Reporting Summary [file 42003_2022_4044_MOESM5_ESM.pdf]

nature research

Corresponding author(s): Rachel Chan

Last updated by author(s): Sep, 19, 2022

Reporting Summary

Nature Research wishes to improve the reproducibility of the work that we publish. This form provides structure for consistency and transparency in reporting. For further information on Nature Research policies, see our Editorial Policies and the Editorial Policy Checklist.

Statistics

For all statistical analyses, confirm that the following items are present in the figure legend, table legend, main text, or Methods section.

n/a	Confirmed	
☐	☑	The exact sample size (*n*) for each experimental group/condition, given as a discrete number and unit of measurement
☐	☑	A statement on whether measurements were taken from distinct samples or whether the same sample was measured repeatedly
☐	☑	The statistical test(s) used AND whether they are one- or two-sided<br>*Only common tests should be described solely by name; describe more complex techniques in the Methods section.*
☑	☐	A description of all covariates tested
☐	☑	A description of any assumptions or corrections, such as tests of normality and adjustment for multiple comparisons
☐	☑	A full description of the statistical parameters including central tendency (e.g. means) or other basic estimates (e.g. regression coefficient) AND variation (e.g. standard deviation) or associated estimates of uncertainty (e.g. confidence intervals)
☐	☑	For null hypothesis testing, the test statistic (e.g. *F*, *t*, *r*) with confidence intervals, effect sizes, degrees of freedom and *P* value noted<br>*Give P values as exact values whenever suitable.*
☑	☐	For Bayesian analysis, information on the choice of priors and Markov chain Monte Carlo settings
☑	☐	For hierarchical and complex designs, identification of the appropriate level for tests and full reporting of outcomes
☑	☐	Estimates of effect sizes (e.g. Cohen's *d*, Pearson's *r*), indicating how they were calculated

Our web collection on statistics for biologists contains articles on many of the points above.

Software and code

Policy information about availability of computer code

Data collection	-FACS data was collected by a CytoFlex flow cytometer (Beckman Coulter, CA, USA).<br>-Immunofluorescence images were acquired by a Carl Zeiss LSM 710 or 800 inverted confocal microscope.
Data analysis	-FACS data was analyzed by the FlowJo Software version 10.5.0 (Tree Star Inc, RO, USA).<br>-Immunofluorescence images were analyzed by the Zeiss LSM ZEN 2019 software (Carl Zeiss, Munich, Germany)<br>-Western blotting were quantified by the Quantity One software.<br>-Statistical analysis was performed with GraphPad Prism version 8.

For manuscripts utilizing custom algorithms or software that are central to the research but not yet described in published literature, software must be made available to editors and reviewers. We strongly encourage code deposition in a community repository (e.g. GitHub). See the Nature Research guidelines for submitting code & software for further information.

Data

Policy information about availability of data

All manuscripts must include a data availability statement. This statement should provide the following information, where applicable:

- Accession codes, unique identifiers, or web links for publicly available datasets
- A list of figures that have associated raw data
- A description of any restrictions on data availability

All source data underlying the graphs presented in the main and supplementary figures are provided in the supplementary information file “Source data”. Uncropped images of the western blotting are presented in the supplementary Figure 5. Other data supporting the findings of this study are available from the corresponding author upon reasonable request.

Field-specific reporting

Please select the one below that is the best fit for your research. If you are not sure, read the appropriate sections before making your selection.

- [x] Life sciences
- [ ] Behavioural & social sciences
- [ ] Ecological, evolutionary & environmental sciences

For a reference copy of the document with all sections, see nature.com/documents/nr-reporting-summary-flat.pdf

Life sciences study design

All studies must disclose on these points even when the disclosure is negative.

Sample size	For in vitro study, at least 3 human primary endometrial cells were used in each group. For in vivo study, at least 3 mice were included in each group.Sample size were determined by our previous experiments.
Data exclusions	Data were only excluded for failed experiments. The reasons for failed experiments included wrong conditions and microbial contamination.
Replication	Reproducibility was tested by performing independent replications per experiment as indicated in the manuscript.
Randomization	Human primary endometrial stromal cells and animals were randomly allocated into each experimental groups.
Blinding	No blinding was performed.

Reporting for specific materials, systems and methods

We require information from authors about some types of materials, experimental systems and methods used in many studies. Here, indicate whether each material, system or method listed is relevant to your study. If you are not sure if a list item applies to your research, read the appropriate section before selecting a response.

Materials & experimental systems

n/a	Involved in the study	
☐	☒	Antibodies
☒	☐	Eukaryotic cell lines
☒	☐	Palaeontology and archaeology
☐	☒	Animals and other organisms
☐	☒	Human research participants
☒	☐	Clinical data
☒	☐	Dual use research of concern

Methods

n/a	Involved in the study	
☒	☐	ChIP-seq
☐	☒	Flow cytometry
☒	☐	MRI-based neuroimaging

Antibodies

Antibodies used	All of the antibodies were purchased from commercial sources with validation data sheets. The information on all antibodies used in the study was listed in supplementary tables.
Validation	All antibodies validation data sheets were provided by the manufactures.

Animals and other organisms

Policy information about studies involving animals; ARRIVE guidelines recommended for reporting animal research

Laboratory animals	Day 19 prepubertal C57BL/6J female mice and 6-week-old C57BL/6J male mice were provided by Centre of Comparative Medicine Research at The University of Hong kong.
Wild animals	N/A
Field-collected samples	N/A
Ethics oversight	All experimental procedures performed in this study were approved by the Committee on Use of Live Animals in Teaching and Research, The University of Hong kong, Hong kong. (CULTRA:5529-20)

Note that full information on the approval of the study protocol must also be provided in the manuscript.

Human research participants

Policy information about studies involving human research participants

Population characteristics	Endometrial tissues were obtained from 27 women aged 41 to 52 years, who underwent abdominal hysterectomy for benign non-endometrial pathologies
Recruitment	Pre-menopausal women with regular menstrual cycle and not taken any hormonal therapy for at least 3 months were recruited. A written consent was signed by each patient after detailed counselling prior to participation of the study.
Ethics oversight	Ethical approval was obtained from the Institutional Review Board of The University of Hong kong/Hospital Authority Hong kong West Cluster (UW20-465) and The Institutional Review Board of the University of Hong kong-Shenzhen Hospital (2018 94)

Note that full information on the approval of the study protocol must also be provided in the manuscript.

Flow Cytometry

Plots

Confirm that:

- [x] The axis labels state the marker and fluorochrome used (e.g. CD4-FITC).
- [x] The axis scales are clearly visible. Include numbers along axes only for bottom left plot of group (a 'group' is an analysis of identical markers).
- [x] All plots are contour plots with outliers or pseudocolor plots.
- [x] A numerical value for number of cells or percentage (with statistics) is provided.

Methodology

Sample preparation	Human primary endometrial stromal cells (detailed description available in the methods section)
Instrument	CytoFlex flow cytometer (Beckman Coulter, CA, USA)
Software	FlowJo Software version 10.5.0 (Tree Star Inc, RO, USA)
Cell population abundance	Cell sorting was not performed.
Gating strategy	Single cell gating: FSC-height versus SSC-height, followed by FSC-height versus FSC-width.<br>Isotype matched antibodies were used to determine the negative and positive boundaries.

- [ ] Tick this box to confirm that a figure exemplifying the gating strategy is provided in the Supplementary Information.
